# Elevated granulocyte-colony stimulating factor and hematopoietic stem cell mobilization in Niemann-Pick type C1 disease

**DOI:** 10.1016/j.jlr.2021.100167

**Published:** 2022-01-08

**Authors:** Anouk G. Groenen, Anouk M. La Rose, Mengying Li, Venetia Bazioti, Arthur F. Svendsen, Niels J. Kloosterhuis, Albertina Ausema, Alle Pranger, M. Rebecca Heiner-Fokkema, Klary E. Niezen-Koning, Tom Houben, Ronit Shiri-Sverdlov, Marit Westerterp

**Affiliations:** 1 Department of Pediatrics, University Medical Center Groningen, University of Groningen, Groningen, The Netherlands; 2 Department of Genetics and Cell Biology, School of Nutrition and Translational Research in Metabolism (NUTRIM), University of Maastricht, Maastricht, The Netherlands; 3 European Research Institute for the Biology of Ageing, University Medical Center Groningen, University of Groningen, Groningen, The Netherlands; 4 Department of Laboratory Medicine, University Medical Center Groningen, University of Groningen, Groningen, The Netherlands

**Keywords:** cholesterol/trafficking, storage diseases, neutrophils, macrophages/monocytes, animal models, inflammation, splenomegaly, hematopoietic stem cells, 7-KC, 7-ketocholesterol, BM, bone marrow, BMT, bone marrow transplantation, C-triol, cholestan-3β,5α,6β-triol, cDNA, complementary DNA, cKit+, cKit-positive, G-CSF, granulocyte-colony stimulating factor, GMP, granulocyte macrophage progenitor, HSC, hematopoietic stem cell, HSPC, hematopoietic stem and progenitor cell, *IL-23a*, interleukin-23a, LDLR, LDL receptor, Lin^−^, lineage-negative, LT-HSC, long-term hematopoietic stem cell, M-CSF, macrophage-colony stimulating factor, *Mcp-1*, monocyte chemoattractant protein-1, MPP, multipotential progenitor, NPC1, Niemann-Pick type C1, *Npc1*^*nih*^, Niemann-Pick type C1^*nih*^, *Nr4a1*, nuclear receptor subfamily 4, group A, member 1, oxLDL, oxidized LDL, RBC, red blood cell, Sca1^+^, stem cells antigen-1-positive, ST-HSC, short-term hematopoietic stem cell, WBC, white blood cell, WT, wild-type, WTD, Western-type diet

## Abstract

Niemann-Pick type C1 (NPC1) disease is a progressive lysosomal storage disorder caused by mutations of the *NPC1* gene. While neurodegeneration is the most severe symptom, a large proportion of NPC1 patients also present with splenomegaly, which has been attributed to cholesterol and glycosphingolipid accumulation in late endosomes and lysosomes. However, recent data also reveal an increase in the inflammatory monocyte subset in the *Npc1*^*nih*^ mouse model expressing an *Npc1* null allele. We evaluated the contribution of hematopoietic cells to splenomegaly in NPC1 disease under conditions of hypercholesterolemia. We transplanted *Npc1*^*nih*^ (*Npc1* null mutation) or *Npc1*^*wt*^ bone marrow (BM) into *Ldlr*^*−/−*^ mice and fed these mice a cholesterol-rich Western-type diet. At 9 weeks after BM transplant, on a chow diet, the *Npc1* null mutation increased plasma granulocyte-colony stimulating factor (G-CSF) by 2-fold and caused mild neutrophilia. At 18 weeks after BM transplant, including 9 weeks of Western-type diet feeding, the *Npc1* mutation increased *G-csf* mRNA levels by ∼5-fold in splenic monocytes/macrophages accompanied by a ∼4-fold increase in splenic neutrophils compared with controls. We also observed ∼5-fold increased long-term and short-term hematopoietic stem cells (HSCs) in the spleen, and a ∼30–75% decrease of these populations in BM, reflecting HSC mobilization, presumably downstream of elevated G-CSF. In line with these data, four patients with NPC1 disease showed higher plasma G-CSF compared with age-matched and gender-matched healthy controls. In conclusion, we show elevated G-CSF levels and HSC mobilization in the setting of an *Npc1* null mutation and propose that this contributes to splenomegaly in patients with NPC1 disease.

Niemann-Pick type C (NPC) disease is a progressive lysosomal storage disorder with an estimated incidence of 1:100,000–120,000 among newborns (1, 2), caused by an autosomal recessive mutation in the *NPC1* (95% of the cases; Online Mendelian Inheritance in Man no.: 257220) or *NPC2* gene. While functions of NPC1 and NPC2 are different, NPC disease caused by mutations in either gene are clinically indistinguishable (3, 4). NPC2 is present on the luminal surface of late endosomes and lysosomes, where it transports cholesterol to NPC1. NPC1 is a transmembrane protein that subsequently transports cholesterol from the late endosomes and lysosomes to the plasma membrane and endoplasmic reticulum (5, 6, 7, 8, 9). Mutations in either gene cause accumulation of glycosphingolipids and cholesterol in late endosomes/lysosomes of all tissues because of their crucial role in transport of cholesterol and sphingolipids out of these organelles (10, 11). The age of onset of NPC disease varies (12). The most severe symptom is neurodegeneration, whereas additional symptoms, such as neonatal jaundice, hepatosplenomegaly, cholestasis, liver, and pulmonary disease, have been reported in a significant number of patients (13). Although neurodegeneration is the most common symptom of NPC disease (14), a high proportion (∼40–50%) of NPC patients presents with splenomegaly during infancy (12, 13). Splenomegaly has been suggested as the first symptom of NPC1 disease before neurological symptoms appear (15). Moreover, in six reported cases of NPC disease, splenomegaly is the only physical symptom (15, 16, 17). Splenomegaly is accompanied by life-threatening effects including increased susceptibility to infections and increased risk of rupture (18). While accumulation of cholesterol and glycosphingolipids in late endosomes and lysosomes of macrophages with *Npc1* loss-of-function may contribute to splenomegaly (19, 20), the exact cause for this phenotype is unknown. Studies using antisense oligonucleotides for *Npc1* that mainly cause deletion of this gene in hepatocytes have suggested that *Npc1* deficiency causes extramedullary hematopoiesis (21). Extramedullary hematopoiesis is the result of hematopoietic stem and progenitor cell (HSPC) mobilization from the bone marrow (BM) to liver and/or spleen. As a consequence, these organs resume their fetal hematopoietic function, reflected by stem cell proliferation and production of monocytes and neutrophils, which causes hepatosplenomegaly (22, 23). This is most likely the consequence of a secretion of factors that stimulate the mobilization of HSPCs from the BM, such as granulocyte-colony stimulating factor (G-CSF) (24). HSPC mobilization has been suggested to contribute to hepatomegaly in NPC disease (21). However, this hypothesis has not been tested directly, and it is unclear whether HSPC mobilization might link to splenomegaly in NPC disease. We here investigated whether a loss-of-function mutation of *Npc1* in hematopoietic cells (11) causes splenomegaly and HSPC mobilization. Our previous studies have shown that deficiency of the cholesterol transporters ABCA1 and ABCG1 in monocytes/macrophages, causing plasma membrane and lysosomal cholesterol accumulation, increases HSPC mobilization and plasma G-CSF (25, 26). The increase in plasma G-CSF was aggravated in the setting of hypercholesterolemia on the LDL receptor (LDLR)-deficient (*Ldlr*^*−/−*^) background upon cholesterol-rich Western-type diet (WTD) feeding (25). We anticipated that, similar to studies in mice with *Abca1/Abcg1* deficiency (26), *Npc1* deficiency in macrophages may play a key role in HSPC mobilization. Cholesterol that accumulates in the endolysosomal system mainly originates from oxidized LDL (oxLDL) (27). Therefore, we investigated the effects of *Npc1* loss-of-function on splenomegaly under conditions of high plasma LDL-cholesterol levels in *Ldlr*^*−/−*^ mice fed a WTD. We used plasma from patients with *NPC1* missense mutations to assess human relevance.

## Materials and methods

### Animals

Niemann-Pick type C1^nih^ (*Npc1*^*nih*^), also known as *Npc1*^*m1N*^, heterozygous mice on the C57BL/6 background were intercrossed to generate homozygous *Npc1*^*wt*^ and *Npc1*^*nih*^ littermates (28). Mice homozygous for the *Npc1*^*nih*^ null mutation, hereafter referred to as *Npc1*^*mut*^, show similar phenotypes to human carriers of *NPC1* mutation including lysosomal cholesterol and sphingolipid accumulation, hepatosplenomegaly, and neurologic impairment (29, 30). *Npc1*^*wt*^ and *Npc1*^*mut*^ BM donors were sacrificed at 5 weeks of age, femur and tibia were collected, and BM was harvested. Mice deficient in the LDLR (*Ldlr*^*−/−*^) on the C57BL/6 background (stock no.: 002207) were obtained from Jackson Laboratories (Bar Harbor, ME) and bred inhouse. Mice were housed under standard laboratory conditions with a light cycle of 12 h and ad libitum water and food. Mice were randomly assigned to experimental groups. The number of mice used for each experiment is indicated in the figure legends. All animal studies were approved by the Institutional Animal Care and Use Committee from the University of Groningen under permit number AVD105002015244 and adhered to guidelines set out in the 2010/63/European Union directive.

### Patients

Four patients carrying *NPC1* missense mutations and age-matched and gender-matched controls were included in this study (31). The characteristics of these patients and their age-matched and gender-matched controls are shown in Table 1. In two of four patients, filipin staining was performed and positive. Three patients presented with juvenile onset of NPC1 disease and one patient with adult onset (31). Plasma was collected from these patients and their age-matched and gender-matched controls during a regular visit at the University Medical Center Groningen. The need for formal ethical review was waived by the local ethics committee of the University Medical Center Groningen, since we made use of blood that was drawn regularly during outpatient visits and leftover from diagnostic investigation. The study design was in accordance with the current revision of the Helsinki Declaration.

**Table 1:** Characteristics of NPC1 patients and controls

Patient	Gender	Age at Plasma Investigation (Years)	Control/NPC1 Mutation	Treatment After Plasma Investigation
1	Female	69	Compound heterozygous mutations c.180G>T (p.Gln60His) and c.2849T>G (p.Val950Gly)	None
2	Female	24	Compound heterozygous mutations c.1211G>A (p.Arg404Gln) and c.2861C>T (p.Ser954Leu)	Liver transplantation
3	Female	18	Homozygous mutations c.1918G>A (p.Gly640Arg)	Miglustat
4	Male	17	Compound heterozygous mutations c.346C>T (p.Arg116∗) and c.247A>G (p.Thyr825Cys)	None
5	Female	70	Control	Not applicable
6	Female	26	Control	Not applicable
7	Female	17	Control	Not applicable
8	Male	17	Control	Not applicable

### Bone marrow transplantation

*Ldlr*^*−/−*^ BM recipients were group-housed in individually ventilated cages and received ciprofloxacin (0.1 mg/ml; Fresenius Kabi, Zeist, The Netherlands) in the drinking water for 10 days, starting 1 day prior to irradiation. At 8 weeks of age, *Ldlr*^*−/−*^ BM recipients were irradiated with a lethal dose (9 Gy) using the X-rad 320 irradiator (Precision X-Ray, North Branford, CT). The next day, mice were transplanted with *Npc1*^*wt*^ or *Npc1*^*mut*^ BM by retro-orbital injection of 5 × 10^6^ BM cells. BM donors were 5 weeks of age. Mice were allowed to recover for 3 weeks after bone marrow transplantation (BMT). After the recovery period, mice were transferred to conventional open-top cages. Mice were fed a chow diet for 9 weeks after BMT (catalog no.: V1554; Ssniff Spezialdiäten GmbH, Soest, Germany). Subsequently, mice were fed a WTD (50% carbohydrates, 20% proteins, 21% fat consisting of 20% milk fat and 1% corn oil, 0.15% cholesterol; D12079B; Research Diets, New Brunswick, NJ) for 9 weeks.

### White blood cell counts and flow cytometry

Blood samples were collected by tail bleeding into EDTA-coated tubes and kept on ice. Total white blood cell (WBC) counts were measured using the Medonic CD620 hematology analyzer (Boule Medical, Spanga, Sweden). For flow cytometry, samples were kept at 4°C for the whole procedure unless stated otherwise. Red blood cells (RBCs) were lysed for 5 min (BD Pharm Lyse; BD Bioscience, Franklin Lakes, NJ), and WBCs were centrifuged, washed, and resuspended in HBSS (0.1% BSA and 0.5 mM EDTA). To assess monocytes, monocyte subsets, and neutrophils, cells were stained with a cocktail of antibodies: CD45-APC-Cy7 (catalog no.: 557659; BD Biosciences, Franklin Lakes, NJ), CD115-PE (catalog no.: 135506; BioLegend, San Diego, CA), and Ly6C/G-PercP-Cy5.5 (catalog no.: 561103; BD Biosciences) for 30 min on ice in the dark. Monocytes were identified as CD45^hi^CD115^hi^ and further separated into Ly6C^lo^ and Ly6C^hi^ subsets. Neutrophils were identified as CD45^hi^CD115^lo^Ly6G^hi^. To assess apoptosis, cells were subsequently stained with Caspase3/7 Red Reagent (Sartorius) in RPMI 1640 medium (Gibco) supplemented with 10% FCS and 1% penicillin-streptomycin for 30 min at 37°C.

Spleens were isolated and mashed on a 40 μm strainer. The femur and tibia were collected. BM was harvested and mashed on a 40 μm strainer. For both BM and spleen samples, RBCs were lysed for 2 min on ice. WBCs were centrifuged, washed, and resuspended in HBSS (0.1% BSA and 0.5 mM EDTA). Total WBC counts in spleen and BM were measured using the Medonic CD620 hematology analyzer. Monocytes, monocyte subsets, and neutrophils in BM and spleen were assessed by flow cytometry using the staining described previously for blood WBCs. To assess HSPCs in BM and spleen, cells were stained with a cocktail of antibodies (all from BioLegend, San Diego, CA): Sca1-BV421 (catalog no.: 108127), cKit-PE (catalog no.: 105808), CD150-PECy7 (catalog no.: 115914), CD48-AF647 (catalog no.: 103416), B220-A700 (catalog no.: 103231), CD11b-A700 (catalog no.: 101222), CD3-A700 (catalog no.: 100216), Ly6C/G-A700 (catalog no.: 108422), and Ter119-A700 (catalog no.: 116220) for 30 min on ice in the dark. HSPCs were identified as lineage-negative (Lin^−^), stem cells antigen-1-positive (Sca1^+^), and cKit-positive (cKit^+^) and further separated into long-term hematopoietic stem cells (LT-HSCs), short-term hematopoietic stem cells (ST-HSCs), and multipotential progenitor (MPP) cells. LT-HSCs were identified as Lin^−^Sca1^+^cKit^+^CD48^−^CD150^+^, ST-HSCs as Lin^−^Sca1^+^cKit^+^CD48^−^CD150^−^, and MPPs as Lin^−^Sca1^+^cKit^+^ CD48^+^CD150^−^.

All samples were analyzed on an LSRII (BD Biosciences), running FACSDiVa software (BD Biosciences). Data were analyzed using FlowJo software (FlowJo, Ashland, OR).

### Plasma total cholesterol and lipoprotein cholesterol distribution

Blood samples were collected from mice. Plasma was separated by centrifugation, and cholesterol levels were measured using an enzymatic kit (catalog no.: 113009910026; Diasys Diagnostic Systems, Holzheim, Germany) with Cholesterol FS standard (catalog no.: 113009910030; Diasys Diagnostic Systems) for the calibration curve. Lipoprotein cholesterol distribution was measured by fast performance liquid chromatography using a system containing a PU-4180 pump with a linear degasser and UV-4075 UV/VIS detectors (Jasco, Tokyo, Japan). Pooled plasma samples (n = 15–16 mice per pool) were injected onto a Superose 6 Increase 10/300 GL column (GE Healthcare, Hoevelaken, The Netherlands) and eluted at a constant flow rate of 0.31 ml/min in PBS (pH 7.4). Cholesterol was measured in line by addition of cholesterol reagent at a constant flow rate of 0.1 ml/min using an additional PU-4080i infusion pump (Jasco, Tokyo, Japan). Data acquisition and analysis were performed using ChromNav software (version 1.0; Jasco, Tokyo, Japan).

### Granulocyte colony-stimulating factor ELISAs

Blood samples were collected from mice or patients carrying *NPC1* mutations and their age-matched and gender-matched controls. Plasma was separated by centrifugation, and granulocyte colony-stimulating factor (G-CSF) levels were measured in plasma using ELISA kits (for mouse: MCS00; for humans: DCS50; R&D Systems, Minneapolis, MN) according to the manufacturer's instructions.

### Plasma oxysterol analysis

Blood samples were collected from mice, and plasma was separated by centrifugation. Approximately 100 μl internal standard for 7-ketocholesterol (7-KC)-d7 or cholestan-3β,5α,6β-triol (C-triol)-d7 and 50 μl butylhydroxytoluene in methanol (40 g/l) were added to 50 μl plasma and mixed for 10 min. Two extractions were performed using methyl tert-butyl ether. Then, 1 ml water was added to a combined sample of two fractions and mixed for 1 min. The two fractions were separated from water and dried using nitrogen. Subsequently, 3 ml hexane and 100 μl Sylon-BTZ (BSA:trimethylchlorosilane:*N*-trimethylsilyimidazole 3:2:3) were added, and the whole sample was incubated for 10 min, resulting in formation of TMS derivates from 7-KC or C-triol. These were analyzed with gas chromatography-tandem MS (Agilent 7000B triple quadrupole; 7890A GC) using positive chemical ionization with 5% ammonia in methane as reaction gas and a nonpolar DB-5MS (15 m × 0.250 mm × 1.00 μm) column (Agilent). Approximately 10 μl sample was injected according to the solvent vent approach at 50°C for 0.45 min. Subsequently, the temperature increased to 300°C with 600°C/min. The oven temperature was 70°C for 2.64 min and increased to 320°C with 40°C/min and hold time 10.5 min. The pressure in the column was 16 psi. The MS-source temperature is 300°C, and the quadrupole is 150°C. The selected mass transitions were C-triol precursor *m/z* 475.4, product *m/z* 457.4; C-triol-d7 precursor *m/z* 482.4, product *m/z* 464.4; 7-KC precursor m/z 473.4, product *m/z* 383.4; and 7-KC-d7 precursor *m/z* 480.5 and product *m/z* 390.5.

### Monocyte assays

Monocytes were isolated from BM of wild-type (WT) mice using CD11b^+^ beads and cultured in DMEM (Gibco) supplemented with 20% L cell conditioned medium, 10% FCS, and 1% penicillin-streptomycin. After 2 h, 5 μg/ml U18666A (catalog no.: 3039-71-2; MilliporeSigma, MO) and 20 μg/ml 7-KC (catalog no.: 566-28-9; MilliporeSigma) were added for 17 h. Cells were collected and lysed in RNeasy lysis buffer. RNA was extracted using an RNeasy mini kit (catalog no.: 74106; Qiagen, Hilden Germany) according to the manufacturer's instructions. Complementary DNA (cDNA) was synthesized using the transcriptor universal cDNA master kit (Roche, Basel, Switzerland). *G-csf* (forward: 5′-GTTCCCCTGGTCAC TGTCA-3′; reverse 5′-TAGGTGGCACACAACTGCTC-3′) mRNA expression levels were assessed by quantitative PCR using Quant Studio 7 Flex Real-Time PCR System (Applied Biosystems, Foster City, CA) and corrected for initial variabilities in RNA quantity using the housekeeping genes *m36B4* and *cyclophilin B*.

### Isolation of splenic monocytes and macrophages

Spleens were isolated and mashed on a 40 μm strainer. RBCs were lysed for 2 min on ice, and WBCs were centrifuged, washed, and resuspended in PBS (0.5% BSA and 2 mM EDTA). First, splenic homogenates were depleted of Ly6G^+^ cells using Ly6G-coated magnetic beads (catalog no.: 130-120-337; Miltenyi Biotec, Bergisch Gladbach, Germany) according to the manufacturer's instructions. CD11b^+^ cells were isolated from splenic homogenates using CD11b-coated magnetic beads (catalog no.: 130-049-601; Miltenyi Biotec) according to the manufacturer's instructions. Cells were lysed in RNeasy lysis buffer, and RNA was extracted using an RNeasy mini kit (catalog no.: 74106; Qiagen) according to the manufacturer's instructions. cDNA was synthesized using the transcriptor universal cDNA master kit (Roche). *G-csf* (for primers, see aforementioned), *Il23a* (forward: 5′-CGCCAAGGTCTGGC TTTTT-3′; reverse: 5′-CGCTGCCACTGCTGACTAGA-3′), *Tnfα* (forward: 5′-GTAGCCCACGTCGTAGCAAAC-3′; reverse: 5′-AGTTGGTTGTCTTTGAGATCCATG-3′), monocyte chemoattractant protein-1 (*Mcp-1*) (forward: 5′-GCTGG AGAGCTACAAGAGGATCA-3′; reverse: 5′-ACAGACCTC TCTCTTGAGCTTGGT-3′), macrophage-colony stimulating factor (*M-csf*) (forward: 5′-GCCTAGGGTGTGATCGTCTC-3′; reverse: 5′-GTAAGGTTCAGGCTCGGTGA-3′), and nuclear receptor subfamily 4, group A, member 1 (*Nr4a1*) (forward: 5′-TTGAGTTCGGCAAGCCTACC-3′; reverse: 5′-GTGTACCC GTCCATGAAGGTG-3′) mRNA expression levels were assessed by quantitative PCR using QuantStudio 7 Flex Real-Time PCR System (Applied Biosystems) and corrected for initial variabilities in RNA quantity using the housekeeping genes *m36B4* and *cyclophilin B*.

### Statistical analysis

All data are presented as mean ± SEM. The two-tailed unpaired *t*-test was used to compare two datasets. The one-way ANOVA with a Bonferroni multiple comparison post-test was used to compare four groups. To assess differences between *NPC1* mutation carriers and their age-matched and gender-matched controls, one-tailed paired *t*-test was used, taking into account data clustering based on age and gender. Group size and statistical test are reported in the figure legend. The criterion for significance was set at *P* < 0.05. Statistical analysis was performed using GraphPad Prism 5 (GraphPad Software, Inc, San Diego, CA).

## Results

### Effects of hematopoietic *Npc1* loss-of-function on myeloid cells in *Ldlr*^*−/−*^ mice

**Fig. 1:**
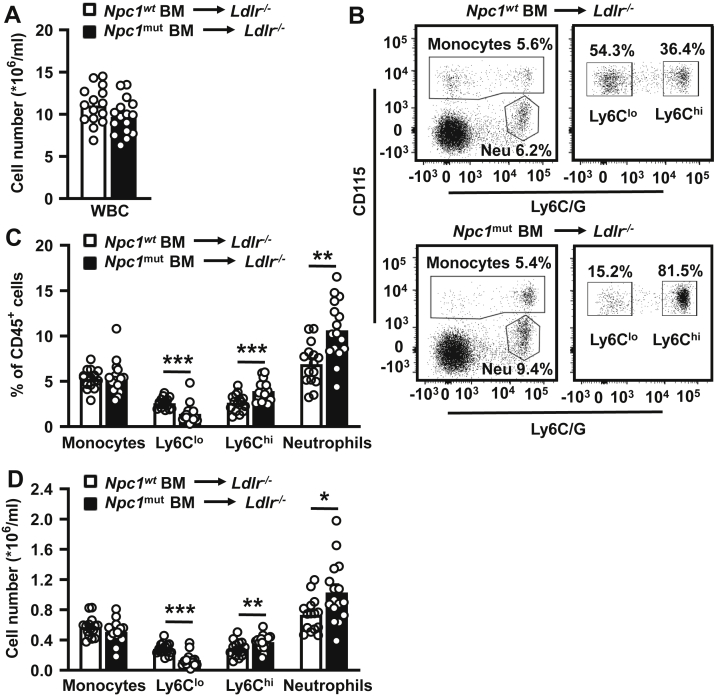
Effects of the *Npc1* loss-of-function mutation in hematopoietic cells on blood myeloid cells in *Ldlr*^*−/−*^ mice fed chow diet. Mice deficient in the LDLR (*Ldlr*^*−/−*^) were transplanted with Niemann-Pick type C1 wild-type (*Npc1*^*wt*^) or Niemann-Pick type C1 mutant (*Npc1*^*mut*^) BM and fed a chow diet. At 5 weeks after BMT, total WBC counts were measured using the Medonic CD620 hematology analyzer (A), and blood monocyte and neutrophil levels were assessed by flow cytometry (B–D). B: Representative flow cytometry plots of monocytes and neutrophils as percentage of total CD45^+^ leukocytes and Ly6C^lo^ and Ly6C^hi^ monocyte subsets as percentage of CD115^+^ monocytes. Neu denotes neutrophils. C and D: Total monocytes, monocyte subsets, and neutrophils as a percentage of total CD45^+^ leukocytes (C) or as total cell number, after correction for WBC (D). n = 15–16 mice per genotype. Data are shown as mean ± SEM. ∗*P* < 0.05, ∗∗*P* < 0.01, ∗∗∗*P* < 0.001 by *t*-test.

Splenomegaly in patients with NPC1 disease can be due to various mechanisms that are presumably regulated via its role in intracellular cholesterol transport in hematopoietic cells. To investigate the role of hematopoietic *Npc1* in splenomegaly under conditions of high LDL-cholesterol, we transplanted BM from mice with a homozygous *Npc1* null mutation (*Npc1*^*mut*^) or from *Npc1* WT (*Npc1*^*wt*^) mice (controls) into *Ldlr*^*−/−*^ mice. At 5 weeks after BMT, when mice were fed a chow diet, we observed no effects of hematopoietic *Npc1* mutation on total WBC counts (Fig. 1A), suggesting no difference in BM reconstitution between the groups of mice, which was similar to a previous study using the exact same approach (28). Hematopoietic *Npc1* loss-of-function did not affect total blood monocytes in *Ldlr*^*−/−*^ mice but decreased Ly6C^lo^ monocytes by ∼55% and increased proinflammatory Ly6C^hi^ monocytes by ∼38% and blood neutrophils by ∼41% (Fig. 1B, D). While effects of the *Npc1* mutation on blood neutrophils have not been reported previously, the observations on blood monocytes were in line with previous findings and already observed in mice with the same *Npc1* mutation at 5 weeks of age, the same age of the BM donors (32).

**Fig. 2:**
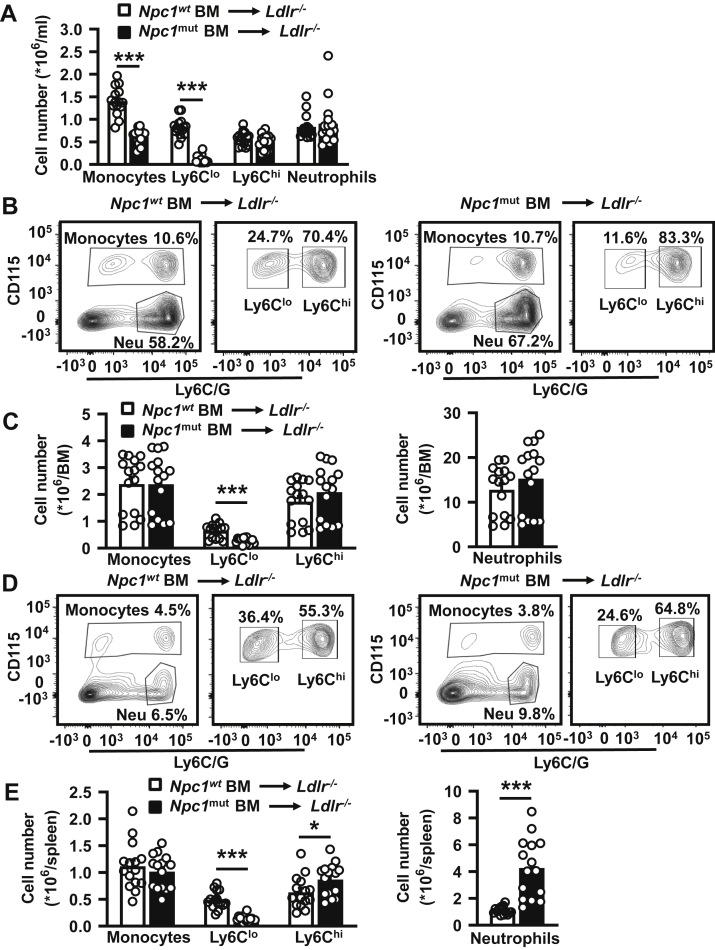
Effects of the *Npc1* loss-of-function mutation in hematopoietic cells on blood, splenic, and bone marrow myeloid cells in WTD-fed *Ldlr*^*−/−*^ mice. *Ldlr*^*−/−*^ mice were transplanted with *Npc1*^*wt*^ or *Npc1*^*mut*^ BM and fed a chow diet for 9 weeks, followed by WTD for 9 weeks. A: Total monocytes, monocyte subsets, and neutrophils in blood from mice fed WTD for 4 weeks, assessed by flow cytometry. BM (B and C) and spleen (D and E) were collected after 9 weeks of WTD feeding, and monocytes and neutrophils were assessed by flow cytometry. B and D: Representative flow cytometry plots of monocytes and neutrophils as percentage of total CD45^+^ leukocytes and Ly6C^lo^ and Ly6C^hi^ monocyte subsets as percentage of CD115^+^ monocytes in BM (B) and spleen (D). Neu denotes neutrophils. Total monocytes, monocyte subsets, and neutrophils in BM (C) and spleen (E). n = 15–16 mice per genotype. Data are shown as mean ± SEM. ∗∗∗*P* < 0.001 by *t*-test.

WTD feeding increased blood monocytes in *Npc1*^*wt*^ BMT *Ldlr*^*−/−*^ mice, in line with previous findings (33, 34), but, unexpectedly, not in *Npc1*^*mut*^ BMT *Ldlr*^*−/−*^ mice (Fig. 2A and supplemental Fig. S1). Surprisingly, the *Npc1* mutation also did not longer affect blood neutrophil levels in *Ldlr*^*−/−*^ mice fed WTD (Fig. 2A and supplemental Fig. S1). The ∼48% decrease in blood monocytes in WTD-fed *Npc1*^*mut*^ BMT compared with *Npc1*^*wt*^ BMT *Ldlr*^*−/−*^ mice was reflected by a decrease in the Ly6C^lo^ monocyte subset (Fig. 2A and supplemental Fig. S1). In addition, hematopoietic *Npc1* loss-of-function mutation decreased plasma cholesterol levels, mainly in WTD-fed *Ldlr*^*−/−*^ mice (Table 2), in line with previous studies (28, 35). The decrease in plasma cholesterol was reflected by a reduction in the VLDL-cholesterol and LDL-cholesterol in *Ldlr*^*−/−*^ mice fed a WTD for 9 weeks (supplemental Fig. S2). Similar to findings in blood, also in BM of WTD-fed *Npc1*^*mut*^ BMT *Ldlr*^*−/−*^ mice, Ly6C^lo^ monocytes were decreased, whereas Ly6C^hi^ monocytes and neutrophils were not affected compared with WTD-fed *Npc1*^*wt*^ BMT *Ldlr*^*−/−*^ mice (Fig. 2B, C). In spleen, we observed a similar reduction in Ly6C^lo^ monocytes of WTD-fed *Npc1*^*mut*^ BMT *Ldlr*^*−/−*^ mice, whereas Ly6C^hi^ monocytes and neutrophils were increased compared with WTD-fed *Npc1*^*wt*^ BMT *Ldlr*^*−/−*^ mice (Fig. 2D, E). The increase in neutrophils was ∼4-fold (Fig. 2D, E).

**Table 2:** Plasma cholesterol levels in *Npc1*^*wt*^ or *Npc1*^*mut*^ BMT *Ldlr*^*−/−*^ mice fed chow diet or WTD

Diet	*Npc1*^wt^ BMT *Ldlr*^*−/−*^	*Npc1*^mut^ BMT *Ldlr*^*−/−*^
Chow diet (7 weeks)	226.0 ± 4.9 mg/dl	205.4^a^ ± 5.1 mg/dl
WTD (4 weeks)	815.5 ± 30.3 mg/dl	613.8^b^ ± 21.4 mg/dl
WTD (9 weeks)	1146.1 ± 67.2 mg/dl	753.7^b^ ± 29.7 mg/dl

Plasma cholesterol levels were determined using an enzymatic kit. n = 15–16 mice per genotype.

a *P* < 0.01.

b *P* < 0.001, by *t*-test.

Collectively, the *Npc1* mutation in hematopoietic cells decreased blood, BM, and splenic Ly6C^lo^ monocytes in *Ldlr*^*−/−*^ mice fed chow diet or WTD. The *Npc1* mutation increased blood Ly6C^hi^ monocytes and neutrophils only in chow diet, but not WTD-fed *Ldlr*^*−/−*^ mice, while increasing splenic Ly6C^hi^ monocytes and neutrophils in WTD-fed *Ldlr*^*−/−*^ mice. Of all changes, the decrease in Ly6C^lo^ monocytes (∼74%) and the increase in splenic neutrophils were most pronounced (∼4-fold).

### Effects of the *Npc1* mutation in hematopoietic cells on inflammatory gene expression in splenic monocytes and macrophages of WTD-fed *Ldlr*^*−/−*^ mice

**Fig. 3:**
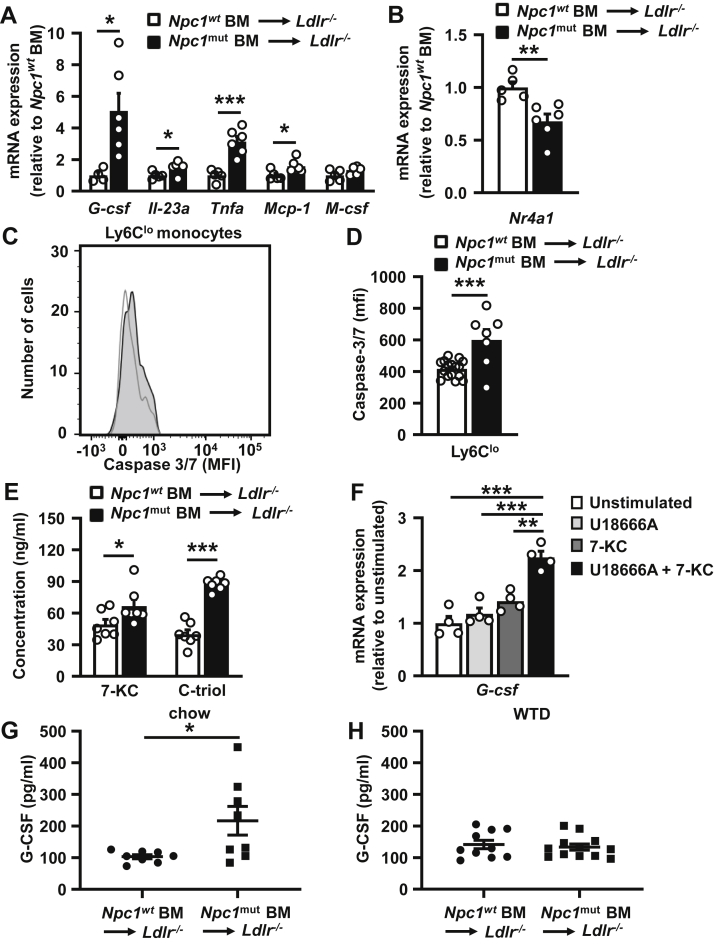
Effects of Npc1 on oxysterols in mice and inflammatory cytokine expression in monocytes and macrophages. A–G: Mice were the same as in Fig. 2. A and B: Spleens were collected after 9 weeks of WTD feeding, Ly6G^−^CD11b^+^ cells were isolated, and RNA was extracted. mRNA expression of *G-csf*, *Il23a*, *Tnfα*, *Mcp-1*, and *M-csf* (A) and *Nr4a1* (B) was assessed in splenic Ly6G^−^CD11b^+^ cells and is expressed relative to mRNA expression in splenic Ly6G^−^CD11b^+^ cells of *Npc1*^*wt*^ BM donors. n = 5–6 mice per genotype. Nr4a1 (Nur77 or NGFIB), nuclear receptor subfamily 4, group A, member 1. C and D: Cleaved caspase 3/7 expression in blood Ly6C^lo^ monocytes was assessed by flow cytometry at 4 weeks of WTD feeding. Representative flow cytometry plots of cleaved caspase 3/7 expression in Ly6C^lo^ monocytes are shown (C) and quantified (D). n = 7–16 mice per genotype. E: Plasma 7-KC and C-triol levels were assessed in *Ldlr*^*−/−*^ mice fed WTD for 9 weeks. n = 7 mice per genotype. F: BM monocytes were isolated from wild-type mice and treated with or without U18666A (5 μg/ml) and with or without 7-KC (20 μg/ml) for 17 h. The mRNA expression of *G-csf* was assessed and is expressed relative to the unstimulated condition. n = 4. G and H: Plasma G-CSF levels were assessed in *Ldlr*^*−/−*^ mice fed chow diet for 9 weeks (G) or fed WTD for 9 weeks (H). Each data point represents one mouse. G: n = 8 mice per genotype; H: n = 10–12 mice per genotype. Data are shown as mean ± SEM. ∗*P* < 0.05, ∗∗*P* < 0.01, ∗∗∗*P* < 0.001 by *t*-test (A–E, G, and H) or one-way ANOVA with Bonferroni post-test (F).

Because of the pronounced effects of the hematopoietic *Npc1* loss-of-function on splenic neutrophils, we characterized the splenic myeloid cell population further. G-CSF production in monocytes and macrophages acts on the granulocyte macrophage progenitors (GMPs) to stimulate neutrophil production, whereas M-CSF acts on GMPs to stimulate the production of monocytes (36, 37, 38). We thus isolated splenic monocytes and macrophages (Ly6G^−^CD11b^+^ cells) and measured *G-csf* and *M-csf* mRNA expression, as well as expression of other proinflammatory cytokines that may be increased in NPC1 disease (28, 39, 40, 41). The *Npc1* mutation increased *G-csf* mRNA expression by ∼5-fold, while not affecting *M-csf*, and increasing *Tnfα* and *Mcp-1* (Fig. 3A). While the increased *G-csf* mRNA expression likely explains the expansion of the splenic neutrophil population, the increase in *Tnfα* and *Mcp-1* suggests an overall increase in inflammation, in line with previous studies (39, 41, 42), and potentially contributing to the increase in Ly6C^hi^ monocytes (43). In addition, we observed that the *Npc1* mutation increased interleukin-23a (*IL-23a*) mRNA expression (Fig. 3A). IL-23 regulates G-CSF levels, by activating the differentiation of naïve T-cells into T_h_17 cells that stimulate production of G-CSF (36). The increase in IL-23 may thus have contributed to the elevated *G-csf* mRNA expression in mice with the *Npc1* mutation. We also observed that the *Npc1* mutation decreased mRNA expression of *Nr4a1* (*Nur77* or *NGFIB*) in splenic monocytes/macrophages (Fig. 3B). Since *Nr4a1* is crucial for Ly6C^lo^ monocyte survival (44, 45), this decrease could explain the decrease in the Ly6C^lo^ monocyte population. We further assessed this by measuring pro-apoptotic cleaved caspase-3/7 in Ly6C^lo^ monocytes. We found that the *Npc1* mutation in hematopoietic cells increased cleaved caspase-3/7 in blood Ly6C^lo^ monocytes (Fig. 3C, D), suggestive of increased apoptosis. This observation is consistent with a decrease in the Ly6C^lo^ monocyte population.

Collectively, the increase in splenic neutrophils in *Ldlr*^*−/−*^ mice with the *Npc1* mutation in hematopoietic cells may be the consequence of increased *G-csf* mRNA expression in splenic monocytes/macrophages, whereas the decrease in *Nr4a1* mRNA expression likely accounts for the decrease in Ly6C^lo^ monocytes. The increase in caspase-3/7 suggests this is downstream of apoptosis.

### NPC1 inhibition increases *G-csf* mRNA expression in monocytes stimulated with 7-KC

We then investigated the mechanism for the increase in *G-csf* mRNA in splenic monocytes/macrophages of *Ldlr*^*−/−*^ mice with the *Npc1* mutation in hematopoietic cells. Previous studies have shown that hematopoietic *Npc1* loss-of-function increases plasma 7-KC and C-triol levels in WTD-fed *Ldlr*^*−/−*^ mice, similar to findings in NPC1 disease patients (35, 46). We replicated this finding in our study (Fig. 3E). The increase in inflammation in NPC1 disease has been attributed to increased oxidative stress downstream of 7-KC accumulation (35), presumably mediated by reactive oxygen species and NF-κB activation (47, 48, 49). We investigated whether 7-KC could account for the increase in *G-csf* mRNA in monocytes and macrophages with *Npc1* loss-of-function. In the unstimulated condition, *G-csf* mRNA expression in BM monocytes was ∼20-fold higher than in BM-derived macrophages (results not shown). We thus used BM monocytes for our experiments and incubated these with 7-KC, while inhibiting NPC1 using the U18666A compound. 7-KC increased *G-csf* mRNA expression in monocytes treated with U18666A (Fig. 3F). These data suggest that the increase in plasma 7-KC may account for the increased *G-csf* mRNA expression in splenic CD11b^+^ monocytes in WTD-fed *Ldlr*^*−/−*^ mice with hematopoietic *Npc1* loss-of-function.

### *Npc1* mutations increase plasma G-CSF levels in *Ldlr*^*−/−*^ mice fed chow diet

We then investigated whether an increase in splenic monocyte/macrophage *G-csf* mRNA expression would translate into an increase in plasma G-CSF levels in the setting of the *Npc1* mutation. While this was indeed the case in *Ldlr*^*−/−*^ mice fed chow diet, hematopoietic *Npc1* loss of function did not affect plasma G-CSF levels in *Ldlr*^*−/−*^ mice fed WTD (Fig. 3G, H). Since monocytes produce G-CSF, we attribute this seeming discrepancy to the decrease in blood monocytes in WTD-fed, but not in chow diet-fed *Npc1*^*mut*^ BMT *Ldlr*^*−/−*^ mice (Figs. 1, 2A). Consistently, the *Npc1* mutation increased blood neutrophil levels in chow diet, but not WTD-fed *Ldlr*^*−/−*^ mice (Figs. 1, 2A).

### Hematopoietic *Npc1* loss-of-function enhances hematopoietic stem cell mobilization in WTD-fed *Ldlr*^*−/−*^ mice

**Fig. 4:**
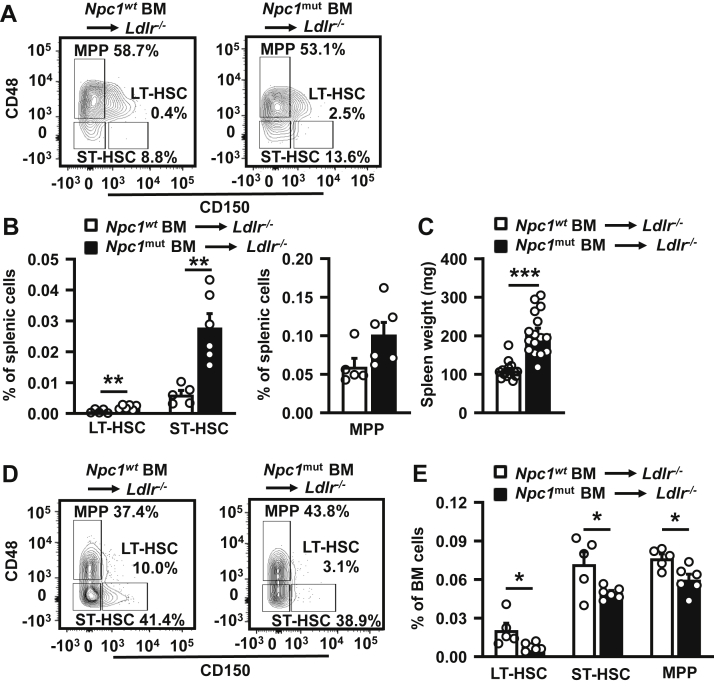
The *Npc1* loss-of-function mutation in hematopoietic cells increases splenic and decreases BM hematopoietic stem cells in WTD-fed *Ldlr*^*−/−*^ mice. After 9 weeks of WTD, spleens (A–C) and BM (D and E) were isolated and LT-HSCs, ST-HSCs, and MPPs were measured by flow cytometry. A: Representative flow cytometry plots of splenic LT-HSCs, ST-HSCs, and MPPs as percentage of Lin^−^, Sca1^+^, cKit^+^ (LSK) cells. B: Quantification of LT-HSCs, ST-HSCs, and MPPs as percentage of total splenic cells. C: Spleen weight. n = 15–16 mice per genotype. D: Representative flow cytometry plots of LT-HSCs ST-HSCs, and MPPs in BM as percentage of LSK cells. E: Quantification of LT-HSCs, ST-HSCs, and MPPs in BM as percentage of total BM cells. A, B, D, and E: n = 5–6 mice per genotype. Data are shown as mean ± SEM. ∗*P* < 0.05, ∗∗*P* < 0.01, ∗∗∗*P* < 0.001 by *t*-test.

G-CSF increases mobilization of stem cells from BM to spleen (26). We thus assessed splenic and BM stem cell populations. The hematopoietic *Npc1* mutation increased LT-HSCs and ST-HSCs in spleen and tended to increase MPPs (Fig. 4A, B), which was accompanied by a 2-fold increase in spleen weight (Fig. 4C). These data are suggestive of HSC mobilization. We also assessed BM stem cell populations. The *Npc1* mutation decreased BM LT-HSC, ST-HSC, and MPP populations (Fig. 4D, E), further supporting HSC mobilization. Together, these data suggest that the *Npc1* mutation in hematopoietic cells induces mobilization of HSCs from BM to spleen. Given that the *Npc1* mutation increases *G-csf* mRNA expression in monocytes and macrophages, and increases plasma G-CSF in chow diet-fed *Ldlr*^*−/−*^ mice, these effects are most likely dependent on G-CSF.

### *NPC1* patients show high plasma G-CSF levels

**Fig. 5:**
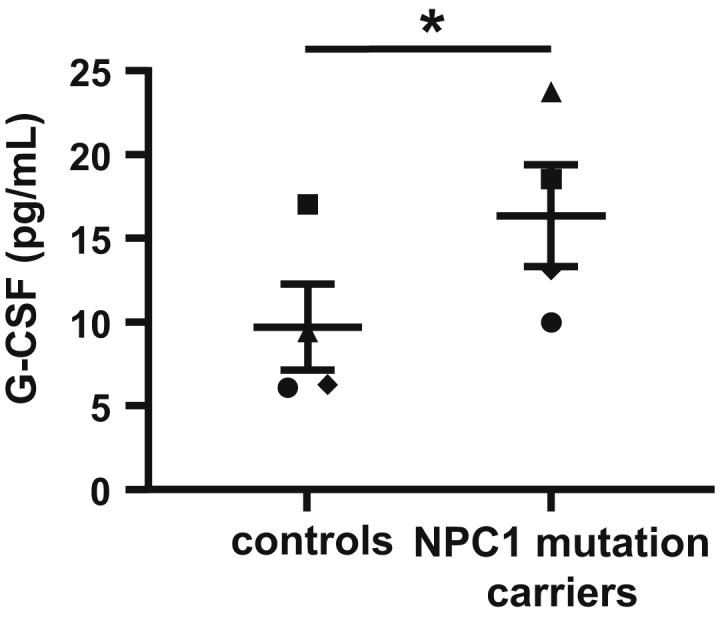
Plasma G-CSF levels in patients with NPC1 disease compared with age-matched and gender-matched healthy controls. Plasma G-CSF levels were assessed in patients carrying *NPC1* mutations and age-matched and gender-matched controls. Each data point represents one patient. The same symbol is used for each *NPC* patient and its age-matched and gender-matched control. n = 4 per group. Data are shown as mean ± SEM. ∗*P* < 0.05 by one-tailed paired *t*-test, taking into account data clustering based on gender and age.

We then evaluated translational relevance of our findings in NPC1 patients. *NPC1* mutations and patient characteristics are listed in Table 1. Plasma samples were drawn from NPC1 patients before any intervention for NPC1 disease. Interestingly, patients carrying mutations in *NPC1* also showed high G-CSF plasma levels compared with gender-matched and aged-matched controls (Fig. 5), suggesting that the findings on G-CSF in mice with the *Npc1* mutation may be relevant for NPC1 disease in humans. Although patient populations were small, it is of interest that the two patients with the highest plasma G-CSF concentration were the ones that eventually received an intervention, either being miglustat, a sphingolipid synthesis inhibitor that has been approved as the first drug specifically targeted for NPC1 disease in 2009 (in Europe), and delays disease progression and improves neurological symptoms (50, 51), or a liver transplantation. These observations perhaps suggest that these patients with the highest plasma G-CSF were the most affected in terms of NPC1 disease.

## Discussion

Approximately 40–50% of NPC1 patients present with splenomegaly during infancy (12, 13). We here identify, using a BMT approach in *Ldlr*^*−/−*^ mice, that the *Npc1*^*nih*^ loss-of-function mutation induces HSC mobilization concomitantly with elevated *G-csf* and *IL-23a* expression in splenic monocytes/macrophages and elevated plasma G-CSF in mice fed a chow diet. Plasma G-CSF levels were also higher in patients with *NPC1* missense mutations. It has been well established that G-CSF induces splenomegaly because of HSC mobilization in mice or humans (18). We thus propose that the increase in G-CSF and HSC mobilization accounts for the splenomegaly in patients with NPC1 disease.

We observed that the *Npc1* loss-of-function mutation increased plasma G-CSF in *Ldlr*^*−/−*^ mice fed chow diet but not WTD. However, on WTD, *Npc1* loss-of-function increased *G-csf* mRNA levels by 5-fold in splenic Ly6G^−^CD11b^+^ monocytes/macrophages. G-CSF has been reported to skew GMPs toward neutrophil production (37). Indeed, the *Npc1* mutation led to a splenic neutrophil expansion of ∼4-fold in WTD-fed *Ldlr*^*−/−*^ mice. In addition, we observed an increase in splenic Ly6C^hi^ monocytes, which we attribute to augmented inflammation (43). Mirroring the findings in spleen, the *Npc1* loss-of-function mutation induced neutrophilia and an increase in Ly6C^hi^ monocytes in blood of chow diet-fed *Ldlr*^*−/−*^ mice. These data suggest that similar mechanisms occur during hematopoiesis early after BMT (in BM) and early extramedullary hematopoiesis in the spleen.

While we cannot exclude an effect of WTD, we propose that plasma G-CSF and blood neutrophils not being elevated by the *Npc1* mutation in *Ldlr*^*−/−*^ mice fed WTD is rather the consequence of the timing of the measurement after BMT than the WTD feeding itself. At 18 weeks after BMT, the *Npc1* mutation decreased blood monocytes by ∼50%, whereas at 9 weeks after BMT, blood monocytes were not affected. Since monocytes produce G-CSF, plasma G-CSF levels no longer being elevated at 18 weeks after BMT may simply have been the consequence of a decrease in blood monocytes. This has likely been preceded by an increase in neutrophil production at the expense of monocyte production by GMPs in mice with the *Npc1* loss-of-function mutation, as shown previously in conditions of high G-CSF (37). Thus, after BMT, high G-CSF in mice with the *Npc1* loss-of-function mutation induced neutrophilia and subsequently a decrease in blood monocytes. The latter resulted in plasma G-CSF no longer being elevated. As a consequence, later after BMT, blood neutrophils were no longer increased.

In addition, we observed that mice with the *Npc1* loss-of-function mutation showed a decrease in Ly6C^lo^ monocytes at all time points after BMT, in blood, BM, and spleen. We attribute this to a decrease in monocyte *Nr4a1* mRNA expression, a survival factor for these cells (44, 45), although further mechanisms for this observation would still need to be examined. Indeed, the *Npc1* loss-of-function mutation increased active caspase-3/7 in Ly6C^lo^ monocytes, which induces apoptosis.

Since we attribute the neutrophil phenotype and the HSC mobilization to elevated G-CSF in *Ldlr*^*−/−*^ mice carrying the *Npc1* mutation, the question arises as to the mechanism that accounts for this finding. We have described previously in the setting of myeloid or dendritic *Abca1* and *Abcg1* deficiency that elevated G-CSF was due to an increase in IL-23 leading to T_h_17 expansion (26), a mechanism originally elucidated by the Ley Laboratory (36). The *Npc1* mutation increased *IL-23a* mRNA expression in splenic Ly6G^−^CD11b^+^ cells by ∼50%, which may not be sufficient to increase *G-csf* mRNA by ∼5-fold, suggesting a direct effect of the *Npc1* mutation on *G-csf* mRNA. The increase in inflammation in NPC1 disease has been attributed to increased oxidative stress downstream of 7-KC accumulation (35). We also found, similar to a previous study (35), that the *Npc1* loss-of-function mutation increased plasma 7-KC in WTD-fed *Ldlr*^*−/−*^ mice. Moreover, our in vitro experiments showed that 7-KC increased *G-csf* mRNA expression in monocytes upon inhibition of Npc1 by U18666A, indicating a role for 7-KC in inducing *G-csf* mRNA expression. 7-KC is a main constituent of oxLDL (52). Interestingly, immunization with heat-killed pneumococci resulting in an increase of plasma IgM antibodies of the E06 idiotype, which neutralize oxLDL, trended strongly toward a decrease in splenomegaly (*P* = 0.06) in WTD-fed *Ldlr*^*−/−*^ mice with the hematopoietic *Npc1* mutation (28). These findings support our idea that oxysterols, main constituents of oxLDL, increase plasma G-CSF levels and splenomegaly. We cannot exclude that other lipid species that accumulate in *Npc1*-deficient monocytes/macrophages, such as bis(monoacylglycero)phosphate (53, 54), may have contributed to the increase in *G-csf* mRNA. We also observed, similar to previous studies (35), that the hematopoietic *Npc1* loss-of-function mutation decreased plasma VLDL/LDL-cholesterol in WTD-fed *Ldlr*^*−/−*^ mice. The latter would be similar to mouse models with hematopoietic or myeloid *Abca1/Abcg1* deficiency or patients with myeloproliferative diseases that are also characterized by a decrease in VLDL/LDL-cholesterol, accompanied by HSPC mobilization and splenomegaly. It has been proposed that the VLDL/LDL-cholesterol uptake by hematopoietic cells drives HSPC proliferation in the spleen, as we recently reviewed (55).

In addition, we found that, similar to *Ldlr*^*−/−*^ mice carrying the *Npc1* mutation in hematopoietic cells on the chow diet, plasma G-CSF was higher in patients with *NPC1* loss-of-function mutations, suggesting human relevance. No abnormalities on WBC populations have been reported in NPC1 disease, except for platelet dysfunction and rare cases of thrombocytopenia (56). It would be of interest to investigate effects of *NPC1* mutations on monocyte subsets, as we observed in mice, in patients with NPC1 disease. This would not necessarily reflect a change in total monocyte numbers.

On the C57BL/6 background, the average life span of mice carrying this *Npc1* mutation is 48.1 ± 5.1 days (57), illustrating the early onset of NPC1 disease. Based on the WBCs after BMT being similar between the groups after BM reconstitution, we anticipate no adverse effects of early onset NPC1 disease to the BM donors. This is also supported by the finding that the decrease in blood Ly6C^lo^ and the increase in Ly6C^hi^ monocyte populations at 5 weeks after BMT is similar to those of the BM donors at 5 weeks of age (32).

In sum, we here elucidate a mechanism that may account for splenomegaly in NPC1 disease, involving increased G-CSF and HSC mobilization. While elevated G-CSF may be disadvantageous in terms of its contribution to splenomegaly, G-CSF stimulates neurogenesis (58), which could counteract the neurodegenerative disease in NPC1 patients. Recent studies have shown that stanol supplementation reduces inflammation in mice with the *Npc1* loss-of-function mutation, including a decrease in inflammatory blood monocytes and hepatic neutrophil infiltration (32, 59), perhaps because of reducing 7-KC. Injections with 2-hydroxypropyl-β-cyclodextrin are currently in phase 2/3 clinical trials for NPC1 disease (NCT02534844) and improve liver function, delay neurodegeneration, and increase life span of *Npc1*^*−/−*^ mice (60, 61, 62) but do not decrease splenomegaly in WTD-fed *Ldlr*^*−/−*^ mice transplanted with BM from mice with *Npc1* loss-of-function (63). A combination therapy of miglustat, 2-hydroxypropyl-β-cyclodextrin, and allopregnanolone decreases splenic T cells, splenic macrophages, and splenic lipid accumulation but does not affect splenomegaly in *Npc1*^*−/−*^ mice on the Balb/c background (20). Effects of miglustat on splenomegaly of *Npc1*^*−/−*^ mice have not been reported (64, 65). Together, these studies suggest that cyclodextrin or miglustat, while delaying neurodegeneration, may not affect splenomegaly in NPC1 disease. We found that 7-KC, which is increased in plasma of NPC1 disease patients, induced *G-csf* mRNA expression upon Npc1 inhibition in monocytes. 7-KC is a main constituent of oxLDL and oxLDL neutralization by increasing plasma IgM antibodies of the E06 idiotype strongly trended toward a decrease in splenomegaly (*P* = 0.06) in a previous study employing the same mouse model of NPC1 disease, where this treatment also improved hepatic inflammation (28). These data indicate a link between oxLDL, 7-KC, G-CSF production, and splenomegaly and suggest E06 as a potential therapy for the peripheral symptoms of NPC1 disease, mainly with regard to the hepatosplenomegaly.

## Data availability

All data are included in the article.

## Supplemental data

This article contains supplemental data.

## Conflict of interest

The authors declare that they have no conflicts of interest with the contents of this article.
