# Supplementary material for: Elevated granulocyte-colony stimulating factor and hematopoietic stem cell mobilization in Niemann-Pick type C1 disease
Source: J Lipid Res. 2022 Jan 8;63(2):100167. doi: 10.1016/j.jlr.2021.100167 (PMC8953690; doi:10.1016/j.jlr.2021.100167)
Supplement: Supplemental Figures S1 and S2 [file mmc1.pdf]

Supplemental Figure 1

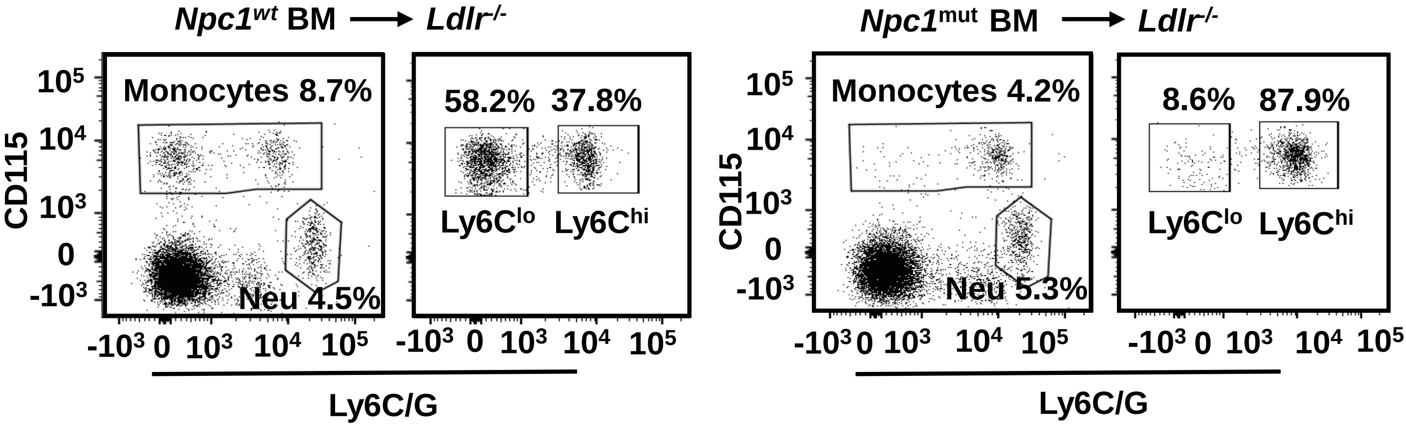

Figure S1. Effects of *Npc1* loss-of-function mutation in hematopoietic cells on blood myeloid cells in *Ldlr$^{-/-}$* mice fed Western-type diet. Mice deficient in the low-density-lipoprotein receptor (*Ldlr$^{-/-}$*) were transplanted with Niemann-Pick Type C1 wild-type (*Npc1^{wt}*) or Niemann-Pick Type C1 mutant (*Npc1^{mut}*) bone marrow (BM) and fed a chow diet for 9 weeks, followed by Western-type diet (WTD) for 9 weeks. Monocyte and neutrophil levels in blood were assessed by flow cytometry at 4 weeks WTD. Representative flow cytometry plots of monocytes and neutrophils as percentage of total CD45$^+$ leukocytes, and Ly6C^{lo} and Ly6C^{hi} monocyte subsets as percentage of CD115$^+$ monocytes on WTD. Neu denotes neutrophils.

Supplemental Figure 2

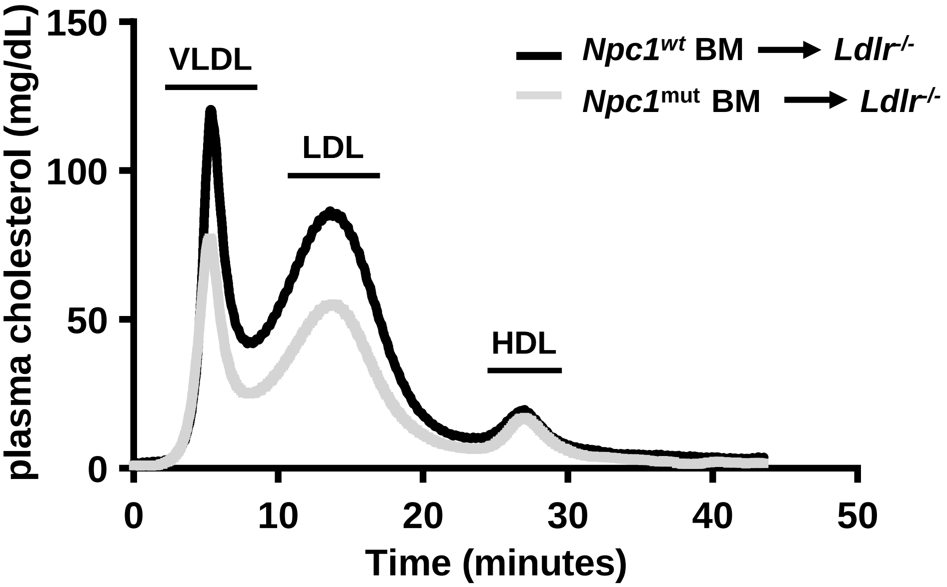

Figure S2. Effects of the *Npc1* loss-of-function mutation in hematopoietic cells on plasma lipoprotein cholesterol distribution in WTD-fed *Ldlr*$^{-/-}$ mice. Mice were the same as in Figure S1. Fast performance liquid chromatography (FPLC) profile of cholesterol on pooled plasma of n=15-16 mice per genotype.
